# Antibacterial activity of selenium nanoparticles/copper oxide (SeNPs/CuO) nanocomposite against some multi-drug resistant clinical pathogens

**DOI:** 10.1186/s12866-025-03743-9

**Published:** 2025-01-20

**Authors:** Ahmed Morad Asaad, Sara A. Saied, Mohammad M. Torayah, N. I. Abu-Elsaad, Samah Mohammed Awad.

**Affiliations:** 1 Medical Microbiology and Immunology Department, Faculty of Medicine, Zagazig University, Zagazig, Egypt; 2 Department of Clinical Pathology, National Liver Institute, Menoufia University, Shibin el Kom, Egypt; 3 Department of Anaesthesia and Intensive care, critical care unit, Faculty of Medicine, Menoufia University, Shibin el Kom, Egypt; 4 Physics Department, Faculty of Science, Zagazig University, Zagazig, Egypt; 5 Clinical microbiology and immunology department, National liver institute, Menoufia University, Shibin el Kom, Egypt

**Keywords:** Selenium nanoparticles, Copper oxide nanoparticle, Multi-drug resistant bacteria, Nanomedicine

## Abstract

**Background:**

Recent advances in nanomedicine have derived novel prospects for development of various bioactive nanoparticles and nanocomposites with significant antibacterial and antifungal properties. This study aims to investigate some characteristics of the novel Se-NPs/Cu_2_O nanocomposite such as morphological, physicochemical, and optical properties, as well as to assess the antibacterial activity of this fabricated composite in different concentrations against some MDR Gram-positive and Gram-negative clinical bacterial isolates.

**Methods:**

The Se-NPs/Cu_2_O nanocomposite was fabricated using the chemical deposition method. The fabricated nanocomposite was fully characterized by X-Ray diffraction analysis (XRD), fourier transforms infrared spectroscopy (FTIR), and transmission electron microscope (TEM). The antimicrobial activity of Se-NPs/Cu_2_O was investigated using the standard broth microdilution method. The fabricated Se-NPs/Cu_2_O nanocomposites were detected as stable and highly crystallized nanospheres with an average size of 98.6 nm.

**Results:**

The Se-NPs/Cu_2_O nanocomposite showed a potent antimicrobial activity with MIC values ranged from 6.25 to 12.5 µg/ml for Gram-positive isolates, and 25 to 50 µg/ml for gram-negative isolates. The bactericidal activity was higher for gram-negative isolates with MBC/MIC ratios of 1–2 µg/ml for gram-negative, versus 8 µg/ml for gram positive pathogens.

**Conclusion:**

These findings would support further research in development of a novel Se-NPs/Cu_2_O nanocomposite as a promising alternative therapeutic option for improving the quality of patients’ management.

## Introduction

The global crisis of antimicrobial resistance (AMR) poses the largest threat to human health and restrains efforts to control and manage human infections. A great concern has been raised on the alarming prospects for AMR to develop into a perfect storm soon in developing and developed countries [1, 2]. The continuous surge of multi-drug resistant (MDR) and pan-drug resistant (PDR) pathogens as well as the stalled development of new antimicrobials by pharmaceutical industry have been directly linked with the increasing global rate of human mortality due to infections by threating microbes [3]. By 2050, 10 million more people would be expected to die every year than would be the case if resistance was kept to today’s level. Therefore, there is increased demand for the development of alternative strategies to conventional antibiotic therapy. Despite the urgent need for newly developed antimicrobials, the drug pipeline is merely empty due to financial issues and limited resources for pharmaceutical industry to promote discovery of newly promising antibiotics [2, 4].

In the last few decades, recent advances in nanotechnology and nanomedicine have derived novel prospects for development of various bioactive nanoparticles (NPs) with antimicrobial properties. Several experimental studies investigated the antimicrobial potentials of various metal NPs including silver, gold, cerium, copper, iron, selenium, and titanium, as well as silicon and carbon-based nanostructures [5, 6]. Moreover, recent efforts have been made in the fabrication of many novel bioactive nanocomposites with significant antibacterial and antifungal properties [7–12].

Among metal NPs, selenium nanoparticles (Se-NPs) have gained much interest due to their great potential in biomedical technology and engineering. Selenium is an essential trace element which plays a vital role in the antioxidant defense systems for protection against oxidative stress. Several experimental studies and technical reports found that Se-NPs have efficient in-vitro and in-vivo antimicrobial activity, low toxicity, and excellent bioavailability compared to other metal nanoparticles [13].

Out of various metal oxides, cuprous oxide (Cu_2_O) is inexpensive, stable in various solutions, and widely used antibacterial structure. The bactericidal activity of Cu_2_O is based on releasing Cu^+ 1^ ions, which induces bacterial cell damage because of cell membrane disruption and RNA leakage [6, 10].

Metal oxides, such as CuO, TiO2, ZnO and Fe3O4, have garnered significant interest for packaging applications due to their distinctive chemical and physical properties, including antibacterial activity, thermal stability, and low toxicity. In the last few years, nanocomposite materials utilizing a polymer matrix of metal and metal oxides NP have attracted significant attention in both research and industry because of their enhanced properties and cost-effectiveness in production [14, 15].

Many previous studies focused on fabrication of nanocomposite materials like Fe3O4@MoS2 core–shell, CuO/Ag-with added zeolite (CAZ), ZnO-CuO NPs/CS, and Fe3O4@SiO2/Schif-base complex of Cu(II) magnetic nanoparticles. The findings of these studies showed clearly the promising antimicrobial activity of fabricated nanocomposites against a variety of human pathogens including bacterial *(S. aures*, *E. faecalis*, *B. cereus*, *B. subtilis*, *E. coli*, and *P. aeruginosa*), and fungal (*Candida* species) isolates [14–17]. Abou Baker and Abbas have successfully fabricated a stable Se-NPs/Cu_2_O nanocomposite with a size of 92.18 nm [18]. This novel nanocomposite showed excellent antimicrobial activity against MDR *H. pylori.* The originality of this study lies in the observation that the synthesized nanocomposite Se-NPs/Cu_2_O would serves as an inhibitor for a wide range of MDR pathogens at low concentrations. There is limited research available on this system in existing literature.

This study aims to investigate some characteristics of the novel Se-NPs/Cu_2_O nanocomposite such as morphological, physicochemical, and optical properties, as well as to assess the antibacterial activity of this fabricated composite in different concentrations against some MDR Gram-positive and Gram-negative clinical bacterial isolates.

## Materials and methods

This in vitro descriptive observational study was conducted at Faculty of Medicine and National liver institute (NLI); a 760-bed tertiary care hospital in Shebin El-Kom, a city in Egypt during the period from January 2023 to January 2024. The research adhered to the Helsinki Declaration principles and approved by the ethical committee (IRB approval number ANET 17 − 2). The study followed the international principles of strengthening the Reporting of Observational Studies in Epidemiology, STROBE [19]. A total of 5 pathogenic MDR bacterial nosocomial isolates were included in this study.

### Bacterial nosocomial isolates

All bacterial isolates were obtained from clinical specimens of hospitalized patients with nosocomial infections, identified based on the criteria of the Centers for Disease Control and Prevention/National Healthcare Safety Network (CDC/NHSN) [20]. The pathogens chosen for this study included MRSA, *Enterococcus faecalis*, *Escherichia coli*, *Klebsiella pneumoniae*, and *Pseudomonas aeruginosa*. These pathogens were predominantly isolated from urinary tract infections, except for MRSA, which was isolated from blood stream infections. Species identification was performed using standard methods [21] and confirmed with the VITEK-2 system (bioMerieux, Marcy-l’Etoile, France) following the manufacturer’s guidelines. Antimicrobial resistance patterns for all isolates were assessed using the reference broth microdilution method in accordance with Clinical and Laboratory Standards Institute (CLSI) guidelines [22]. Methicillin resistance in *S. aureus* strains was detected through PCR amplification of the *mec*A gene as described previously [23]. Multidrug resistance (MDR) was defined as resistance to at least one antimicrobial agent in three or more classes [3].

### Chemicals, reagents, and cultural media

Selenium nanoparticles (Se-NPs), copper (II) sulfate (CuSO_4_, 97%), and ethane-1,2-diamine (99%) were supplied by Sigma-Aldrich (St. Louis, USA). Sodium hydroxide (NaOH, 98%), and ethanol (99.5%) were obtained from Merck Chemicals (Germany). Tropic soy broth/agar and Muller-Hinton broth/agar were purchased from Hi-Media (Mumbai, India).

### Preparation of Cu2O nanospheres

Nano cuprous oxide spheres were prepared in vitro using wet-chemical method [24]. Briefly, 0.4 g of CuSO_4_ was added to 50 ml of distilled water then, 50 mL of aq. NaOH was added drop by drop with a continuous stirring for 10 min to yield a blue precipitate of Cu(OH) _2_. Then, 50 ml of ethane-1,2 diamine was added drop by drop under a continuous stirring at room temperature for 1 h. Finally, the produced participate was washed with a mixture of distilled water and ethanol and dried under a vacuum at 50 ºC for 10 h.

### Fabrication of Se-NPs/Cu2O nanocomposite

The chemical deposition method was used to prepare stable Se-NPs/Cu_2_O nanocomposite [24]. Briefly, 25 mg of Se-NPs was dissolved in 25 mL of acetone in a conical flask, and ultrasonicated for 1 h. Then, a mixture of 25 mg of Cu_2_O dispersed in 25 mL of acetone and water (1:1 by volume) solution was added to the conical flask. Then, the mixture was homogenized using ultrasonication and stirring for 50 min. The mixture was centrifuged, and then the produced Se-NPs/Cu_2_O nanocomposite was purified by washing with acetone and sterile water, and drying for 20 h at -50 °C.

### Characterization of Se-NPs/Cu2O nanocomposite

The fabricated Se-NPs/Cu_2_O nanocomposite was characterized as previously described using X-ray diffraction (XRD) analysis (A PANalytical diffractometer, X’Pert PRO, Netherlands) for analysis of the nanomaterials’ XRD using CuKα X-rays over 20 angle ranged between 5° to 70°, Attenuated Total Reflection Fourier Transform Infrared (ATR-FTIR) measurements using FT-IR spectrophotometer (EQUINOX, Bruker) in the range of 4000 to 450 cm − 1, and transmission electron microscopy (TEM) analysis (JTEM-1230, Japan, JEOL).

### Antimicrobial activity of Se-NPs/Cu2O nanocomposite

The antimicrobial activity of Se-NPs/Cu_2_O nanocomposite was carried out using the standard minimal inhibitory concentration (MIC) and minimal bactericidal concentration (MBC) methods [22].

### The minimum inhibitory concentration

The MIC of Se-NPs/Cu_2_O nanocomposite was determined by the broth microdilution method following the guidelines of the CLSI [22]. From each bacterial isolate, 3–4 colonies were dissolved in a sterile saline, and the suspension was adjusted to achieve a turbidity equivalent to a 0.5 McFarland standard using Densi-CHEK optical device. That adjustment results in a suspension containing approximately 1–2 × 10^8^ CFU/ml. Then, the suspension was diluted by inoculating 1.0 ml of inoculum into 20 ml of Muller Hinton broth, which results in approximately a concentration of 1.0 × 10^6^ CFU/ml.

Double strength MH broth (100 ml) containing 5% dimethyl sulfoxide (DMSO) was dispensed into wells of 96-well microtiter plates. The stock Se-NPs/Cu_2_O nanocomposite solution was diluted and transferred into the first well, and serial dilutions were performed so that concentrations in the range of 100–0.19 µg/ml, (i.e. 100, 50, 25, 12.5, 6.25, 3.12, 1.56, 0.78, 0.39, 0.19 µg/ml) were obtained. To each well, 10 µl of each bacterial suspension (equivalent to a concentration of 5.0 × 10^5^ CFU/m) was added. The set was allowed to incubate aerobically at 37 °C for 24 h. The assay for each of the pathogens was repeated three times to assess and attest reproducibility. MIC was defined as the lowest concentration of each extract that inhibited 90% of visible growth.

### The minimal bactericidal concentration

Wells showing no visible bacterial growth were selected for determining the minimum bactericidal concentration (MBC). To do this, a loopful of each suspension was cultured on Mueller Hinton (MH) agar after homogenization. The cultures were then incubated aerobically at 37 °C overnight. The MBC was determined based on the culture medium where no visible microbial growth was observed. This procedure was repeated three times to ensure consistent results. The antibacterial activity of the Se-NPs/Cu2O nanocomposite was evaluated using the MIC index (MBC/MIC). An MIC index of 1–2 indicates bactericidal activity, while an index of 4–16 suggests bacteriostatic activity [25].

## Results and discussion

### X-ray diffraction (XRD) analysis

**Fig. 1:**
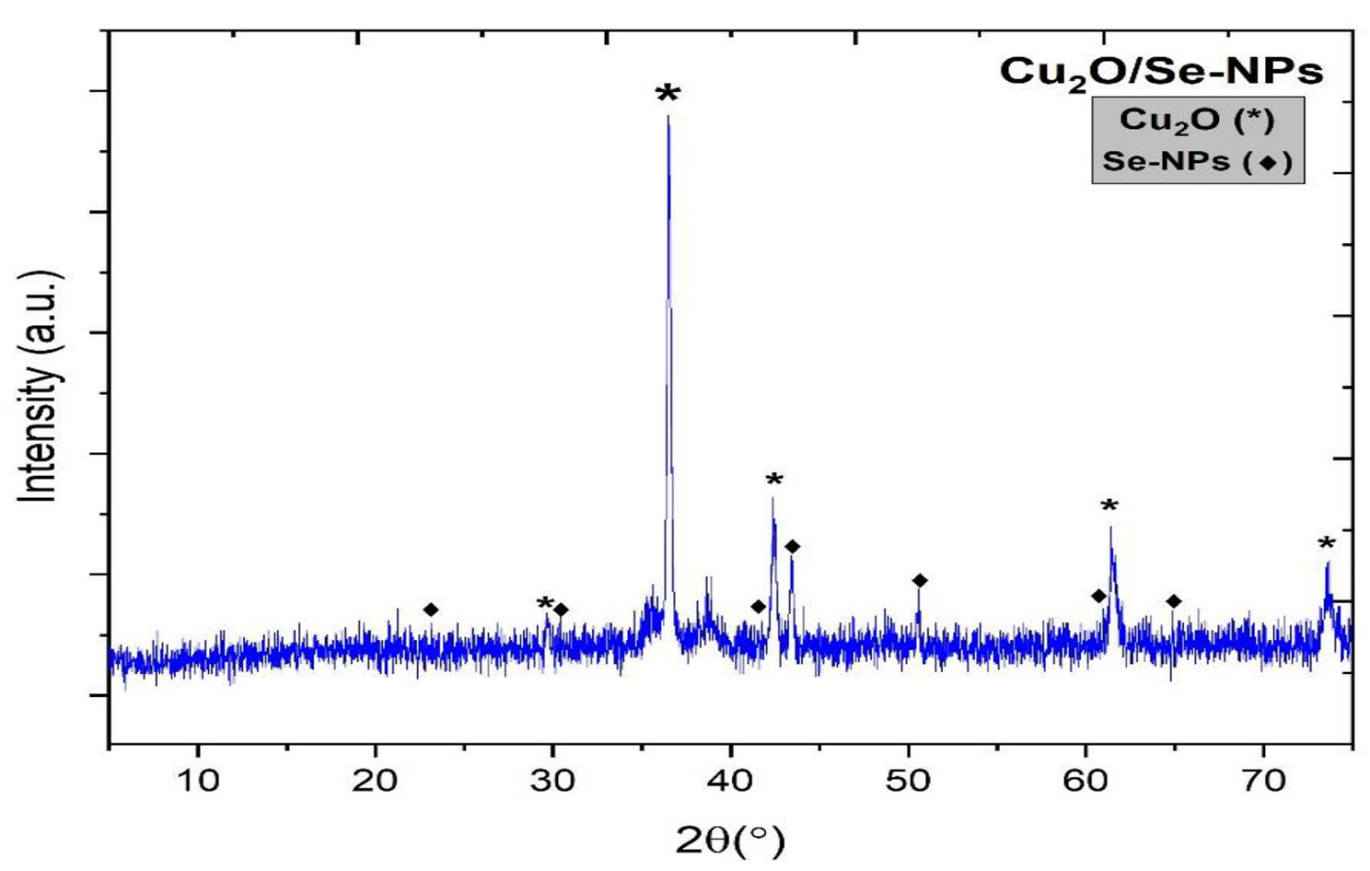
X-Ray diffraction of Se nanoparticles with cuprous oxide

Figure 1 shows the X-Ray diffraction pattern that is used in the determination of crystallographic structures, the nature of crystallinity and the lattice parameter values. The high intense diffraction peaks of Cu_2_O were measured at 2$\:\theta\:=$ 29.6º, 36.4 º, 42.4 º, 61.4 º, and 73.6 º which may be related to the planes of (110), (111), (200), (220), and (311) and to the crystalline cubic phase of Cu_2_O according to ICDD # 00-900-5769 [26]. Furthermore, the diffraction peaks of the hexagonal phase of Se-NPs were ascribed at 2$\:\theta\:=$ 23.1 º, 30.3 º, 41.7 º, 43.3 º, 50.5 º, 60.9 º, and 64.8 º which might assign to the lattice planes of (100), 101), (110), (102), (112), (103), and (210) respectively which agrees with JCPDS Card (No 06-0362) [25].

### Fourier Transform Infrared (ATR-FTIR) measurements

**Fig. 2:**
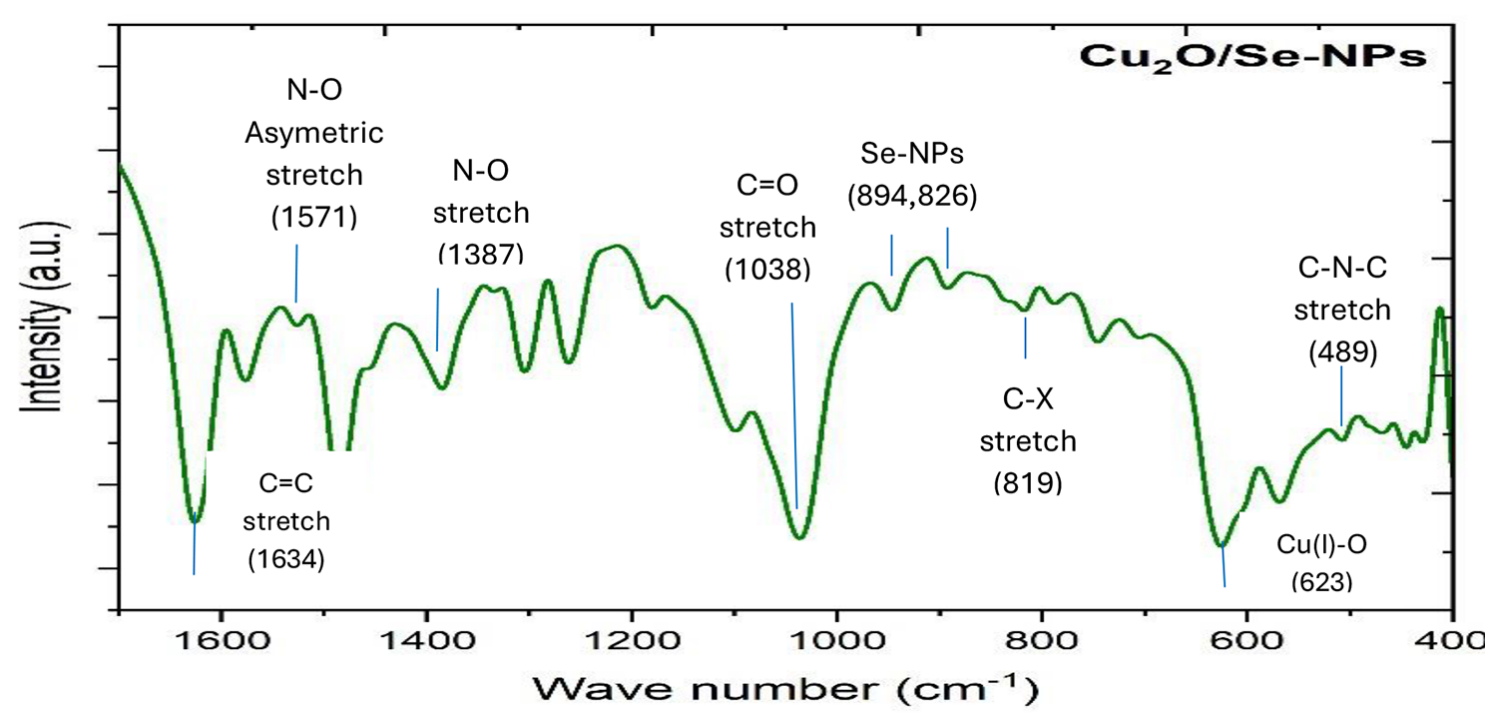
The FTIR pattern of Cu_2_O with Se-NPs

Fourier transforms infrared spectroscopy was used to examine the sample of Se-NPs/Cu_2_O. The main characteristic bands for Cu_2_O were determined at 623 cm^− 1^, 2918 cm^− 1^, and 3433 cm^− 1^ which corresponded to Cu(I)-O vibrations that approved the presence of Cu_2_O nanoparticles, symmetric CH_2_ stretching, and O–H stretching vibration [8, 26–30]. Moreover, the measured bands of Se-NPs at 1038 cm^− 1^ and 1387 cm^− 1^ are attributed to the stretching of the carboxyl group (C = O) or carbonyl group (C-O) and N-O stretching of aliphatic nitro compounds [26], as illustrated in Fig. 2. Another two absorption bands at 826 and 894 cm^− 1^ are due to the presence of Se-NPs. Moreover, A weak absorption band at 489 cm^− 1^ is assigned to C–N–C bending in amines. Meanwhile, C–X stretching in alkyl halides gives a band at 819 cm^− 1^. The band at 1571 cm^− 1^ is attributed to N–O asymmetric stretch nitro compounds. The band at 1627 cm^− 1^ corresponds to C = C stretching vibration of alkene [28, 29].

### Surface topography

**Fig. 3:**
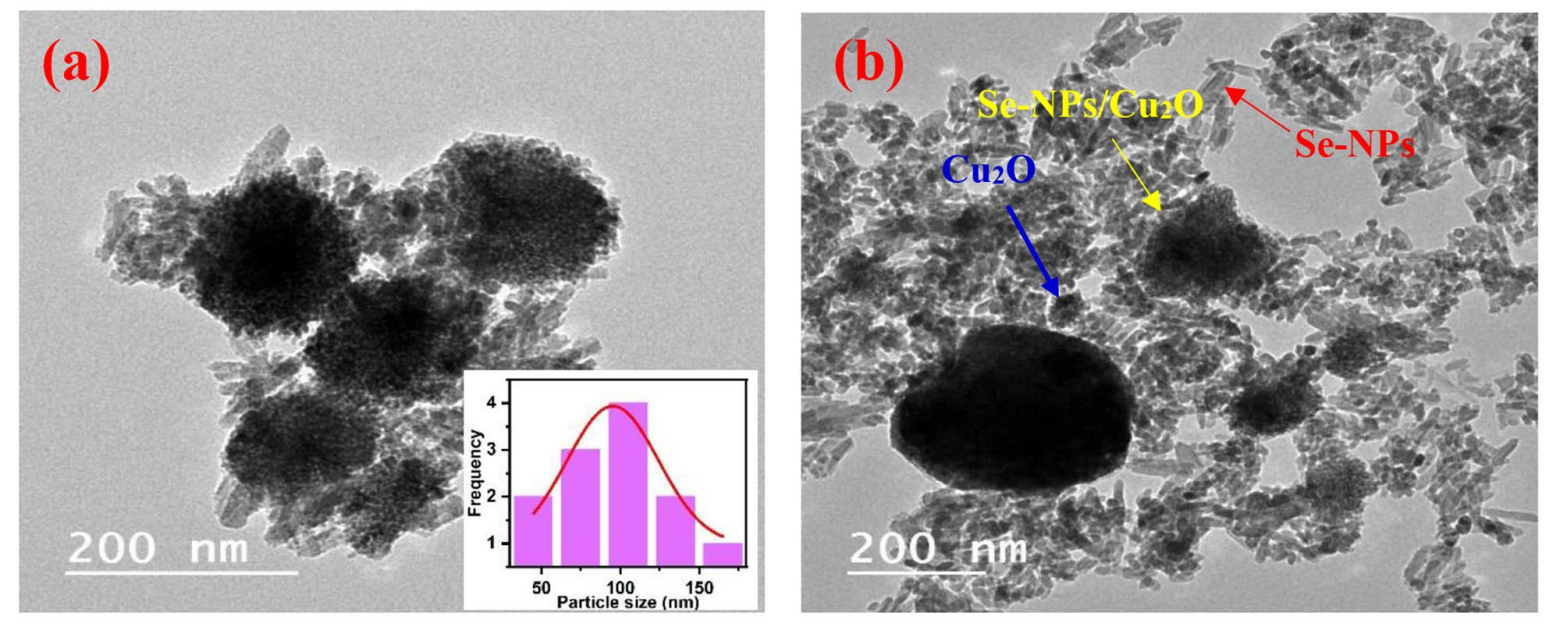
Microscopic images of Se-NPs/Cu_2_O using transmission electron microscope (TEM)

The surface morphology, shape, and size of Se-NPs/Cu_2_O nanocomposite were obtained using a transmission electron microscope (TEM) as shown in Fig. 3. The microscopic images exhibit spherical shapes of Se-NPs/Cu_2_O with an approximately average size of 98.6 nm. The images represent a random distribution of nano-rod shapes of Se-NPs with diameters in the range of 30–80 nm and lengths between 50 and 120 nm. On the other hand, Cu_2_O particles were illustrated as irregular and spherical shapes with diameters between 85 and 160 nm, which well-formed from smaller particles in size. From this observation, the size of uncoated Se- and Cu_2_O - NPs was smaller than coated Se-NPs/Cu_2_O. This is not surprising, as the outer Se NPs integrate with the inner Cu_2_O NPs, and the size of Se-NPs/Cu_2_O nanocomposite has been increased 14 times. In agreement, Abou Baker and Abbas [18] fabricated Se-NPs/Cu_2_O with a size of 92.18 nm. In their study, coating of Se-NPs with Cu_2_O NPs results in an 18 times-increase in size of Se-NPs/Cu_2_O nanocomposite. Many previous experimental studies showed similar results with other metal NPs including Cu_2_O/ZnO, α-Fe_2_O_3_ NPs/ZnO and α-FeOOH/BiOI nanocomposites [30–33].

### Antimicrobial activity of Se-NPs/Cu2O nanocomposite

**Fig. 4:**
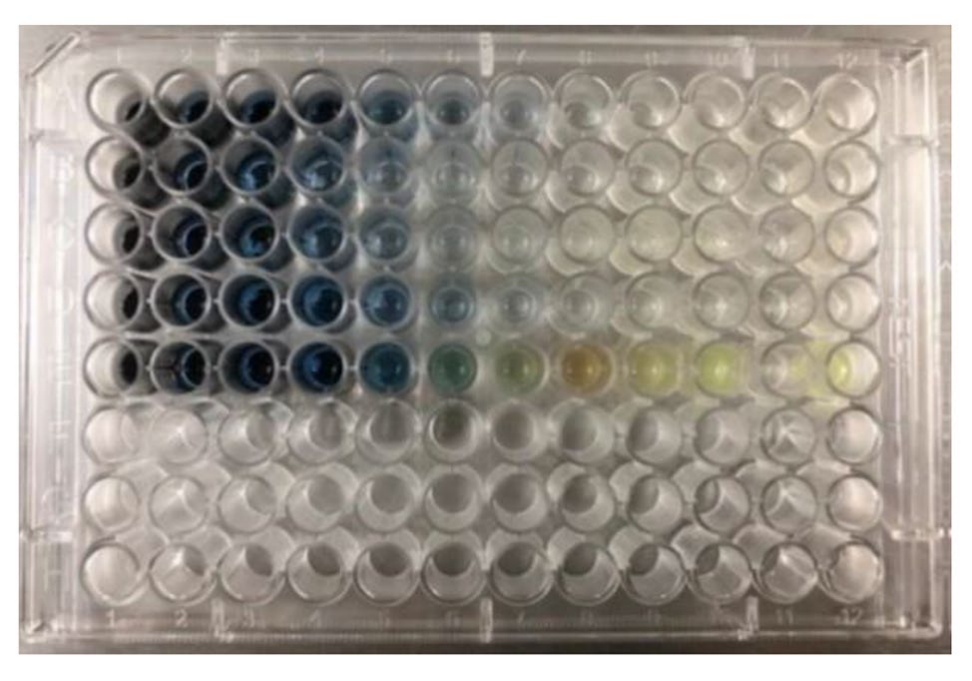
Determination of MIC of Se-NPs/Cu_2_O nanocomposite using broth dilution method The concentrations ranged from 100 to 0.19 µg/ml from left to right. The first vertical row is for negative control. Plate rows from up downwards represent *P. aeruginosa*, *E. coli*, *K. pneumoniae*, *A. baumannii*, and *Staph. aureus*, respectively

The standard broth microdilution method for investigating the antimicrobial activity of Se-NPs/Cu_2_O nanocomposite showed that the MIC values ranged from 6.25 to 12.5 µg/ml for Gram-positive isolates, and 25 to 50 µg/ml for Gram-negative isolates (Table 1; Fig. 4). Unfortunately, there is paucity of information on the bacteriostatic and bactericidal activity of Se-NPs/Cu_2_O nanocomposite. Abou Bakar and Abbas [18] assessed the antimicrobial activity of Se-NPs/Cu_2_O nanocomposite against *H. pylori* using broth dilution method, and found an MIC of 8 µg/ml is sufficient for inhibition of the growth of 100% MDR *H. pylori*. Previous experimental studies have investigated either Cu_2_O- or Se-NPs against a long list of both quality control and clinical bacterial isolates. In the study of Vinu et al. [33] Cu_2_o and Se-NPs exhibited good antimicrobial activity against *P. aeruginosa* with MIC values of 20 ug/ml and 10 ug/ml, respectively. Shehabeldine et al. [25] found that *Klebsiella oxytoca* and *Escherichia coli* were more susceptible to Cu_2_O-NPs with MIC values of 6.25 and 3.12 µg/mL, while for *Staphylococcus aureus* and *Bacillus cereus*, MIC value was 12.5 and 25 µg/mL, respectively. Dang-Bao et al. [34] reported a potent antimicrobial activity of Se nanoparticles and Cu nanoparticles against MRSA with MIC values of 5 µg/L and 40 µg/L, respectively. In this study, the synergistic activity of Se-NPs and Cu_2_O would be promising for further studies focusing on Se-NPs/Cu_2_O nanocomposite as an effective therapeutic option for management of infections caused by MDR bacterial pathogens.

**Table 1:** The MIC, MBC, and MBC/MIC ration of SeNPs/Cu2O nanocompsite against bacterial nosocomial isolates

Bacterial Isolate	MIC (µg/ml)	MBC (µg/ml)	MBC/MIC ratio	Control antibiotic^*^ (µg/ml)
*Staph. aureus*	6.25	50	8	2
*Staph. hominis*	3.12	25	8	1
*E. faecalis*	12.5	100	8	2
*E. coli*	25	50	2	4
*K. pneumoniae*	25	50	2	4
*P. aeruginosa*	50	100	2	8
*A. baumanii*	50	50	1	6

*Control antibiotic: Vancomycin for gram-positive bacteria, and gentamycin for gram-negative bacteria

It is worth noting that the size of Se-NPs/Cu_2_O nanocomposite in this study (98.6 nm) is larger than that reported by Abou Baker and Abbas [18] who fabricated Se-NPs/Cu_2_O with a size of 92.18 nm. Previous studies reported that the size of NPs plays a crucial role in determining the antibacterial activity. Huang et al. [35] reported that Se NPs of 81 nm showed a significant antimicrobial activity against S. aureus with MIC and MBC values of 25 µg/ml and ≤ 50 µg/ml. respectively.

Antimicrobial agents are usually considered bactericidal if the MBC/MIC ratio is ≤ 4 µg/ml and bacteriostatic if > 4 µg/ml [25]. The MBC/MIC ratios of Se-NPs/Cu_2_O nanocomposite fabricated in this work were equal to 8 µg/ml for all gram-positive isolates, indicating bacteriostatic activity while MBC/MIC ratios ranged from 1 to 2 µg/ml for different gram-negative isolates indicating bactericidal activity (Table 1). These finding is not surprising and would be due to the significant difference in bacterial cell wall structure, including abundant pores, a loose peri-plasmic space and a thin layer of peptidoglycan in cell wall of gram-negative bacteria which allow for rapid diffusion of NPs, compared to the strong cell wall with a thick peptidoglycan layer in gram-positive bacteria [36].

This study had some limitations. First, the study was not testing biocompatibility and toxicity of Se-NPs/Cu_2_O nanocomposite. Further experimental studies including MTT assay are necessary for quantitative measurement of cytotoxicity of the fabricated nanoscoposite in response to fibroblast cells viability and proliferation in cell line culture. Second, the study focused solely on the antimicrobial activity of Se-NPs/Cu_2_O nanocomposite against five MDR bacterial pathogens. The findings of this work are promising. Further extensive research is likely required to assess the antimicrobial activity of Se-NPs/Cu_2_O nanocomposite against a broader range of MDR nosocomial bacteria, in addition to exploring its antiviral and antifungal properties.

In conclusion, this work showed successful fabrication of a stable Se-NPs/Cu_2_O nanocomposite using the chemical deposition method. The Se-NPs/Cu_2_O nanocomposite was fully characterized by XRD, ATR-FTIR, and TEM. At the optimal conditions, the fabricated Se-NPs/Cu_2_O nanocomposites were detected as stable and highly crystallized nanospheres with an average size of 98.6 nm. The Se-NPs/Cu_2_O nanocomposite showed a potent antimicrobial activity with a wide range of low MIC values against MDR bacterial clinical isolates with a higher bactericidal activity against gram-negative pathogens (MBC/MIC ratios of 1 to 2 µg/ml for Gram negative pathogens compared to 8 µg/ml for Gram positive strains). These findings are of considerable concern, and would support further research in development of a novel and promising alternative therapeutic option for improving the quality of patients’ management.

## Data Availability

The datasets used and/or analysed during the current study are available from the corresponding author on reasonable request.
